# Solitary Pancreatic Extramedullary Plasmacytoma Diagnosed Through Endoscopic Ultrasound-Guided Fine-Needle Biopsy—The Role of Endoscopic Diagnosis and Multidisciplinary Approach: A Case Report and Literature Review

**DOI:** 10.3390/diagnostics16172680

**Published:** 2026-08-22

**Authors:** Stefano Marletta, Enrico Cavallo, Michele Dello Spedale Venti, Giacomo Bonanno, Fabio Romeo, Alessia La Fauci, Enzo Gallo, Antonio Rizzo, Emanuele Stefano Giovanni d’Amore, Mariangela Novello

**Affiliations:** 1 Division of Pathology, Humanitas Istituto Clinico Catanese, 95045 Catania, Italy; enrico.cavallo@humanitascatania.it (E.C.); michele.dellospedaleventi@humanitascatania.it (M.D.S.V.); antonio.rizzo@humanitascatania.it (A.R.); emanuele.damore@humanitascatania.it (E.S.G.d.); 2 Department of Biomedical Sciences, Humanitas University, 20072 Milano, Italy; 3 Digestive Endoscopy Unit, Humanitas Istituto Clinico Catanese, 95045 Catania, Italy; giacomo.bonanno@humanitascatania.it (G.B.); fabio.romeo@humanitascatania.it (F.R.); 4 Medical Oncology, Humanitas Istituto Clinico Catanese, 95045 Catania, Italy; alessia.lafauci@humanitascatania.it; 5 UOC Pathology, IRCCS National Cancer Institute “Regina Elena”, 00128 Roma, Italy; enzo.gallo@ifo.it (E.G.); mariangela.novello@ifo.it (M.N.)

**Keywords:** extramedullary plasmacytoma, EUS-FNB, molecular testing, multidisciplinary approach

## Abstract

**Background and Clinical Significance:** Primary pancreatic extramedullary plasmacytomas (EMP) are extremely uncommon, representing less than 0.1% of all pancreatic masses and being far more uncommon than secondary involvement by an underlying multiple myeloma. **Case presentation**: A 69-year-old male was found with a solitary pancreatic mass. Endoscopic ultrasound (EUS)-guided fine-needle biopsy (FNB) revealed a monoclonal plasmacytic proliferation with molecularly confirmed KRAS (A146V) and BRAF (G469V) mutations. Multidisciplinary assessment ruled out a secondary localization, thus leading to the ultimate diagnosis of primary pancreatic EMP. **Conclusions**: Reaching a definitive diagnosis of primary pancreatic EMP requires a negative bone marrow biopsy and histological confirmation of the plasma cell nature of the pancreatic process. As for the latter, EUS-FNB may be a pivotal technique to avoid upfront surgical procedures.

## 1. Introduction

Plasma cell neoplasia encompasses a group of hematological diseases driven by a clonal proliferation of post-germinal-center, class-switched, terminally differentiated B cells secreting monoclonal immunoglobulins (“M proteins” or “paraproteins”). Among them, plasma cell myeloma/multiple myeloma is the most common form, characterized by an abnormal clonal expansion of plasma cells in the bone marrow, accounting for more than 10% of the cells [1]. Conversely, plasmacytomas are rare, solitary neoplasms composed of clonal plasma cells with little to no evidence of bone marrow involvement, accounting for 2–5% of all plasma cell neoplasms [2,3]. According to their skeletal or extra-skeletal location, they are further classified as either solitary plasmacytomas of the bone (SPB) or extramedullary plasmacytomas (EMP). The latter are less frequent than SPBs, representing 20–30% of plasmacytomas [4,5]. Although more commonly involving the respiratory tract, EMPs may arise from virtually every organ, including the genitourinary tract, the skin, and the gastrointestinal tract, among others [6]. Within the digestive system, EMPs are often found in the liver or the stomach [7]. Primary pancreatic EMPs are extremely uncommon and can resemble much more frequent pathological processes requiring a surgical approach (i.e., neuroendocrine tumors or pancreatic carcinoma). Thus, their upfront recognition is pivotal to ensuring proper therapeutic management for patients. In the present work, we describe a case of primary pancreatic EMP diagnosed through endoscopic ultrasound (EUS)-guided fine-needle biopsy (FNB) and review the published literature on the topic.

## 2. Case Presentation

### 2.1. Clinical and Imaging Findings

**Figure 1:**
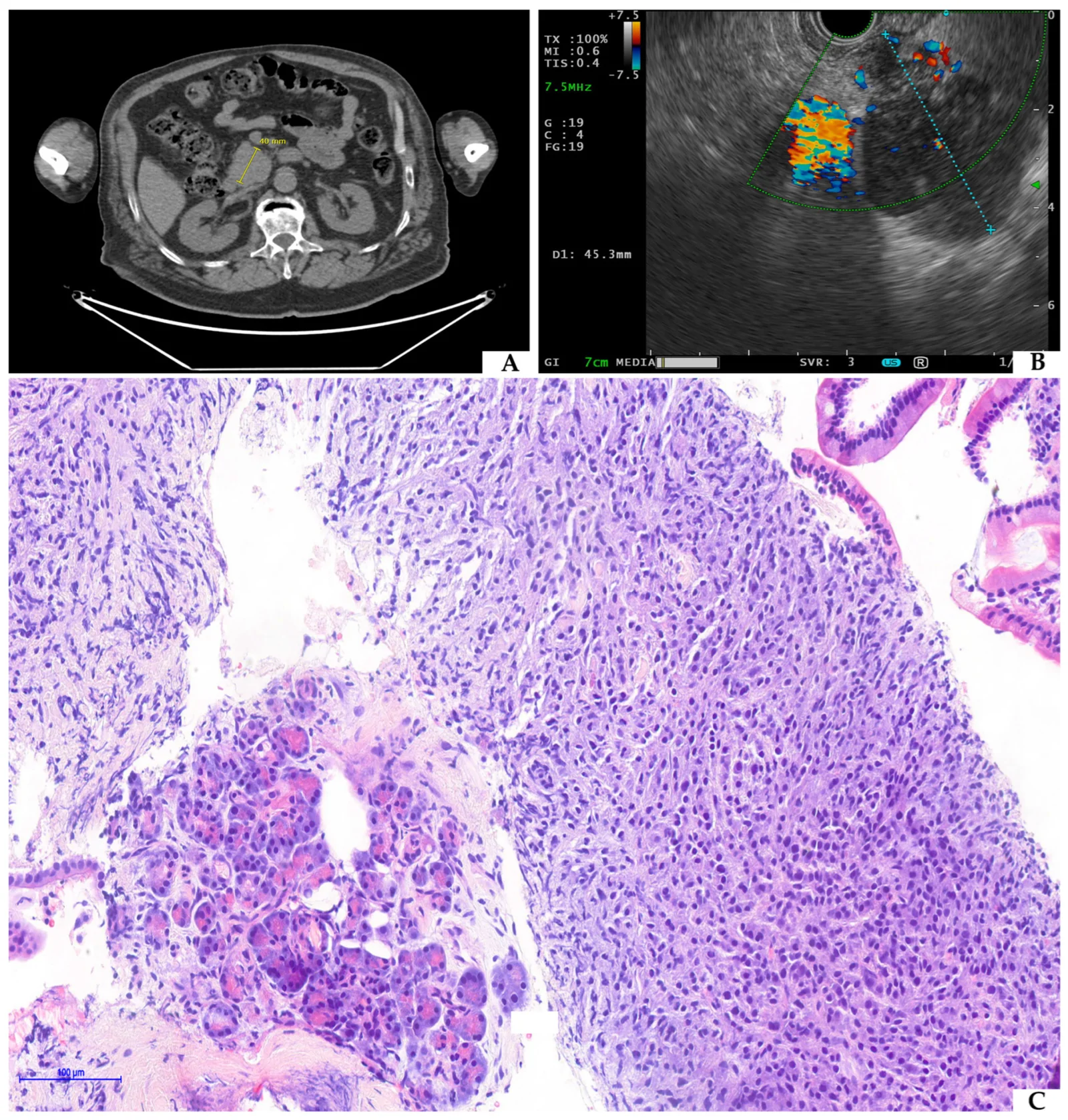
Basal CT scan showing a 40 mm mass with homogeneous density within the head/uncinate process of the pancreas, compressing the right renal vein (**A**). Endoscopic ultrasound examination displaying a 45 mm hypoechoic lesion with internal anechoic areas, irregular margins, and inconsistent color Doppler signal within the pancreatic head/uncinate process (**B**). Hematoxylin and eosin microphotograph documenting a dense proliferation of plasmacytoid-like cellular elements adjacent to normal pancreatic parenchyma (**C**) (original magnification 180X C).

A 69-year-old male was incidentally found with a pancreatic lesion during a post-traumatic ultrasound examination. A subsequent contrast-enhanced CT scan revealed a 40 mm mass with homogenous density in the head/uncinate process of the pancreas, exerting pressure on the right renal vein (Figure 1A). Despite the absence of radiological signs of bile duct dilation or regional lymphadenopathy, the findings raised suspicion for a primary pancreatic neoplasm, such as ductal adenocarcinoma or a neuroendocrine tumor. To further investigate, the patient underwent endoscopic ultrasound-guided fine-needle biopsy (EUS-FNB) using a linear-array video echoendoscope (GF-UCT180, Olympus, Tokyo, Japan) connected to an EU-ME2 Premier Plus (Olympus) ultrasound system. The examination revealed a 45 mm hypoechoic lesion with internal anechoic areas and irregular margins in the uncinate process of the pancreas (Figure 1B). Color Doppler imaging was employed to delineate intervening vessels. Tissue acquisition (TA) was performed with two passes using 22-gauge Acquire (Boston Scientific, Marlborough, MA, USA) needles. The collected samples were fixed in 10% formalin, embedded in paraffin, sectioned at 2–3 μm, and stained with hematoxylin and eosin, with additional unstained sections reserved for further analysis.

### 2.2. Pathological, Immunohistochemical, and Molecular Features and Systemic Workup

Morphological evaluation documented close to normal pancreatic parenchyma, a highly cellular proliferation of plasmacytoid elements with moderate cytological atypia (Figure 1C). Neither coagulative tumor necrosis nor relevant mitotic activity was spotted. Immunohistochemical analysis was performed with the antibodies listed in Table S1, and molecular analysis with Next-Generation Sequencing (NGS) using an Oncomine Comprehensive Assay Plus kit (Thermo Fisher Scientific, Waltham, MA, USA) on an Ion Torrent platform (IonGene Studio S5 Prime, Thermo Fisher Scientific, Waltham, MA, USA).

**Figure 2:**
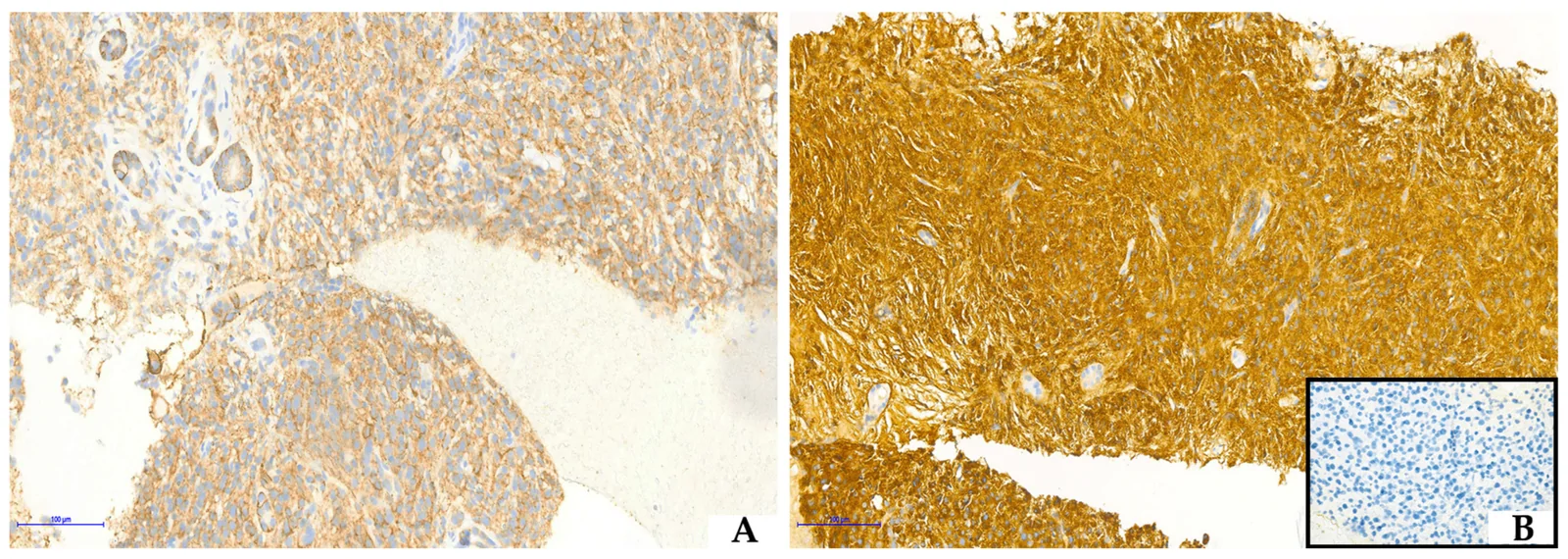
Immunohistochemical analysis highlighting the plasmacytic phenotype of the lesion, which stained positive for CD138, as well as normal entrapped pancreatic ducts (**A**), and showed clonal restriction for Lambda light chains (**B**) and no labeling for Kappa light chains [inset] (original magnifications 200X A and B and 400X inset).

Immunohistochemistry confirmed the plasmacytic phenotype of the neoplastic cells, immunolabeling for CD138 (Figure 2A) and MUM1 but not for CD45, with a clonal restriction for Lambda light chains (Figure 2B), and a low proliferative index (Ki67 10–15%); CD19, CD117, and cyclinD1 were negative, and only focal CD56 positivity was observed.

Moreover, pathogenic point mutations of the *KRAS* (A146V) and *BRAF* (G469V) genes were detected by NGS. An extranodal marginal zone lymphoma (EMZL) with diffuse plasmacytic differentiation and lymphoplasmacytic lymphoma (LPL) were considered in the differential diagnosis. The former was difficult to exclude because EMZL can occasionally show extensive plasma cell differentiation. However, the negativity for CD45 and the focal CD56 positivity of the plasma cells, along with the complete absence of a component of CD20+ B-cells and of molecular alterations typical of EMZL (*TET2*, *TNFRSF14*, *CCR6*, *TBL1XR1*, *NOTCH1-2*), favored the diagnosis of plasmacytoma. An LPL could be more easily ruled by the lack of diffuse bone marrow involvement, IgM blood monoclonal component, and *MYD88* p.L265P mutations [8]. Hence, the combined morphological, immunohistochemical, and molecular findings supported a diagnosis of a mature plasma cell neoplasm. Further investigations were conducted to distinguish between a primary pancreatic EMP and secondary involvement by an undiagnosed plasma cell myeloma. Specific laboratory tests were later performed at an external facility, as they were not available within the hospital service. Namely, serum (heavy and free light-chain assays) and urine analyses (immunofixation and 24 h protein electrophoresis) revealed an IgA paraproteinemia (total IgA 566 mg/dL, reference values 70–400 mg/dL) with a monoclonal Lambda component (SPEP-M spike 0.16 g/dL). Kappa/Lambda serum-free light-chain (sFLC) ratio was 0.55. Hematologic parameters, including leukocyte and platelet counts, as well as renal function and calcium levels, were within normal limits. Bone marrow biopsy showed a slight inversion of the kappa/lambda ratio by immunohistochemistry among resident plasma cells, which accounted for less than 5% of total marrow cellularity—below the diagnostic threshold for multiple myeloma [1]. To note, multi-parameter flow cytometry was not performed. Based on these findings and according to the WHO [1] and International Myeloma Working Group Criteria [3], a final diagnosis of primary pancreatic EMP (with possible minimal marrow involvement) was established.

An 18F-fluorodeoxyglucose positron emission tomography (FDG-PET) scan demonstrated localized tracer uptake in the pancreatic head (SUV max = 6.3), with no evidence of systemic disease. Given the localized presentation, the patient underwent definitive radiotherapy (20 fractions, 40 Gy). To date, 18 months since the initial diagnosis, no evidence of disease was found on follow-up by FDG-PET and laboratory monoclonal component tests.

## 3. Discussion

Solitary EMP is an uncommon hematological condition, representing 2–5% of all plasma cell neoplasia [2]. The occurrence of solitary EMP in the pancreas is exceedingly rare and essentially anecdotal, representing less than 0.1% of primary tumors arising in this organ [9]. Pancreatic involvement by a plasma cell neoplasm has been reported as early as 1947 [10]; however, the case described was most likely attributable to secondary pancreatic infiltration in the context of multiple myeloma. Therefore, the first report of a truly solitary bone-marrow-negative EMP of the pancreas should be set to 1958 [11]. To date, thirteen solitary pancreatic EMPs with a proven negative bone marrow biopsy, including the present one, have been described in the literature (Table 1), all but one being case reports [11,12,13,14,15,16,17,18,19], with a unique small series [7]. Most of them developed in middle-aged males (M:F ratio 1.6:1, mean 60 y.o., median 73 y.o.) and were located in the head of the pancreas (75%).

Beyond its rarity, the present case illustrates the remarkable evolution of the diagnostic approach to pancreatic EMP over recent decades. Historically, most pancreatic EMPs were diagnosed after surgery or even at autopsy. Early reports, including the first well-documented case in 1958 [11], relied on pathological examination of resected specimens, while several later cases were similarly identified only after pancreatic surgery because the lesions were initially interpreted as adenocarcinomas or other primary pancreatic neoplasms. Even in more recent reports [12,16], diagnosis was established only after resection. In contrast, the present case was diagnosed entirely through a minimally invasive approach with EUS-FNB, enabling definitive treatment without surgery. This highlights the transition from a surgery-driven to a pathology-driven diagnostic paradigm and underscores the increasing importance of advanced endoscopic tissue acquisition techniques in the management of rare pancreatic lesions.

The current case also supports the growing role of EUS-FNB over conventional fine-needle aspiration in uncommon pancreatic tumors. Notably, apart from the current case, the definitive pathological diagnosis was reached via EUS in just two other instances [18,19]. In recent decades, TA coupled with EUS has been increasingly employed to access deep intra-abdominal processes such as pancreatic masses [20]. Among these techniques, EUS-FNB has recently emerged as an alternative to fine-needle aspiration (FNA), especially for rare conditions such as metastases, soft tissue lesions, and hematological processes [21,22]. While both techniques provide high diagnostic accuracy, EUS-FNB offers superior preservation of tissue architecture and yields sufficient material for ancillary investigations. This becomes particularly relevant when a hematologic neoplasm is considered in the differential diagnosis, as extensive immunophenotypic and molecular characterization is often required. As for the herein-described solitary pancreatic EMP, EUS-FNB and a global multidisciplinary approach were critical, both for reaching the definitive diagnosis that guided therapeutic management and for obtaining prognostic information. Indeed, ancillary tests and exams such as bone marrow multi-parameter flow cytometry, low-dose CT, and whole-body MRI were unavailable in the present case. However, the global laboratory, radiological, and pathological findings before and after therapy administration confidently support the diagnosis of a solitary pancreatic EMP, advocating reason-guided multidisciplinary management.

Concerning therapy, without EUS-FNB, the patient would have undergone diagnostic pancreaticoduodenectomy, a far too excessive procedure considering the final pathological diagnosis. Conversely, EUS-FNB provided adequate tissue for histopathological examination, immunohistochemistry, and next-generation sequencing, enabling a comprehensive characterization of the lesion. Thus, once a secondary involvement by an underlying systemic multiple myeloma was excluded, the patient was allowed to receive curative radiotherapy, based on the proven radiation sensitivity of EMP [23].

From a clinical perspective, the present report further emphasizes the prognostic importance of distinguishing primary pancreatic EMP from secondary pancreatic involvement by multiple myeloma. While secondary pancreatic localization usually reflects advanced systemic disease and often requires systemic anti-myeloma treatment, true solitary EMPs generally have a more favorable course and may be effectively controlled with local radiotherapy alone [24]. Generally, approximately 70% of patients diagnosed with EMP remain disease-free, with the remaining ones developing either multiple myeloma or multiple extramedullary localizations [1,25]. In this view, among the cases with available follow-up, only a third of patients diagnosed with pancreatic EMP experienced an aggressive clinical behavior [11,17,18], with just two of them dying from disease dissemination [11] or hemodynamic complications (esophageal varices’ bleeding) [17]. The durable disease-free status observed in our patient 18 months after treatment is consistent with previously reported outcomes of localized pancreatic EMP and reinforces the value of accurate staging through bone marrow biopsy, laboratory studies, and PET imaging. On the other hand, the outcome of secondary localizations from systemic multiple myeloma is highly variable and strictly linked to the stage of the underlying disease [9]. The finding of a slight Kappa/Lambda imbalance among bone marrow plasma cells, which account for less than 5% of total marrow cellularity, warrants consideration from a classificatory standpoint. According to current WHO and IMWG criteria, the diagnosis of solitary extramedullary plasmacytoma remains appropriate in the absence of significant marrow plasmacytosis (≥10% clonal plasma cells), myeloma-defining events, or evidence of systemic disease [1,3]. Although the observed marrow findings may suggest minimal occult marrow involvement, they do not meet the diagnostic criteria for plasma cell myeloma and are therefore best interpreted as insufficient evidence of systemic dissemination. In the present case, the lack of CRAB features, the normal serum free light-chain ratio, the absence of additional lesions on FDG-PET, and the sustained complete response following local radiotherapy collectively support classification as a primary pancreatic EMP rather than secondary pancreatic involvement by multiple myeloma.

As for the differential diagnosis, primary EMPs have to be distinguished from lymphomas with plasma cell differentiation. Plasmacytic differentiation may occur in almost all small B-cell lymphomas, but it is particularly prominent in LPL and in rare cases of marginal zone lymphomas [8]. The distinction from LPL is usually straightforward because of the frequent association with IgM paraproteinemia (although approximately 5% of cases are non-IgM-type), bone marrow involvement, and a characteristic admixture of small B lymphocytes, plasmacytoid cells, and plasma cells, often accompanied by increased reactive mast cells. In addition, the presence of the recurrent *MYD88* p.L265P mutation represents a highly characteristic molecular finding [8]. The differential diagnosis is much more challenging when considering an EMZL with extensive plasma cell differentiation. In our case, the morphological and immunophenotypic features favored a diagnosis of plasmacytoma, based on the complete absence of a CD20-positive small B-cell component and the aberrant phenotype of the plasma cells. Furthermore, the presence of IgA paraproteinemia also supported the diagnosis of plasmacytoma/plasma cell neoplasm, as IgA-producing cases have been reported in approximately 25% of extramedullary plasmacytomas [1]. Conversely, although monoclonal paraproteinemia may occur in extranodal marginal zone lymphoma, it is more commonly of IgM type, whereas IgA and IgG paraproteins have been only rarely reported [26]. Additional supportive evidence was provided by the identification of *KRAS* p.A146V and *BRAF* p.G469V mutations, suggesting activation of the mitogen-activated protein kinase (MAPK) pathway. Although MAPK pathway alterations may occur in both plasma cell neoplasms and marginal zone lymphomas, they are more frequently observed in plasma cell neoplasms. Conversely, no mutations involving genes recurrently altered in marginal zone lymphomas, such as *TET2*, *TNFRSF14*, *CCR6*, *TBL1XR1*, *NOTCH1*, and *NOTCH2*, were identified.

Finally, with respect to molecular pathology, to our knowledge, this is the first documented pancreatic EMP harboring concomitant *KRAS* p.A146V and *BRAF* p.G469V mutations. In multiple myeloma, alterations affecting the RAS/RAF/MEK/ERK signaling pathway occur in approximately 40% of newly diagnosed cases [27] and may be associated with a more aggressive clinical course [28], although the prognostic significance remains controversial. Interestingly, relapsed/refractory multiple myeloma with extraosseous extramedullary disease (EMD) almost invariably shows *RAS*/*BRAF* mutations in the extraosseous disease, and it has been hypothesized that these are essential for the development of EMD [29]. Far less is known about solitary extramedullary plasmacytoma, and to our knowledge, no additional data on *RAS* or *BRAF* mutations in pancreatic EMP are currently available. So far, both the frequency of mutations in the RAS/RAF/MEK/ERK pathway and their prognostic implications in primary extraosseous plasma cell neoplasms remain unclear. The increasing availability of MAPK/ERK pathway inhibitors may offer therapeutic opportunities in cases with an unfavorable clinical course [30] and underscores the importance of incorporating molecular testing for these genes into the diagnostic workup of primary extramedullary plasma cell neoplasms. Notably, the *BRAF* G469V mutation identified in the present case belongs to the class II *BRAF* gene mutations, which are generally insensitive to commercially available anti-BRAF inhibitors, which selectively target the V600E (class I) *BRAF* mutation. However, in case of disease progression, the patient could be likely given *MEK* inhibitors or allosteric anti-class II specific agents. Furthermore, another pancreatic EMP testing positive for BRAF V600E by immunohistochemistry has been previously described [18], potentially benefiting from classic anti-class I inhibitors.

## 4. Conclusions

In summary, solitary EMP of the pancreas is extremely rare but should be considered in the management of uncommon pancreatic lesions. Multidisciplinary integration with molecular, laboratory, and hematological data is pivotal for guiding therapeutic choices and ensuring patients achieve the best possible outcomes. Beyond documenting an exceptionally rare neoplasm, this report demonstrates how EUS-FNB-based diagnosis can avoid unnecessary surgery. Furthermore, we have provided the first evidence of KRAS/BRAF pathway alterations in a primary pancreatic EMP, thereby contributing novel biological and potentially therapeutic information to the existing literature.

## Figures and Tables

**Table 1:** Reported cases of solitary pancreatic extramedullary plasmacytoma with available confirmed or presumed negative bone marrow biopsies.

Case n.	Author, Year	Sex	Age	Site	Diagnosis	Genetic Findings	Therapy	FU
1	Richards et al., 1958 [11]	F	77	Head	Autopsy	NA	RT	DOD
2	Deguchi et al., 2004 [12]	M	56	Tail	Surgery	NA	None	NED
3	Wei et al., 2009 [13]	F	53	NA	FNA	NA	CT	NED
4	Wiazzane et al., 2013 [14]	NA	NA	NA	NA	NA	RT	NA
5	Ignjatović et al., 2016 [15]	F	55	Head	Endoscopy biopsy	NA	Surgery	NED
6	Lu et al., 2017 [16]	M	56	Body	Surgery	NA	None	NED
7	Csomor et al., 2019 [17]	NA	52	Head	Endoscopy biopsy	NA	None	DOD
8	Kelley et al., 2022 [7]	NA	NA	NA	NA	NA	NA	NA
9	Kelley et al., 2022 [7]	NA	NA	NA	NA	NA	NA	NA
10	Kelley et al., 2022 [7]	NA	NA	NA	NA	NA	NA	NA
11	Rizk et al., 2024 [18]	M	62	Head	EUS-FNA	BRAF V600E mutation (IHC)	CT, RT	AWD
12	Montenegro et al., 2026 [19]	M	67	Head	EUS-FNB	NA	RT	NED
13	Actual study, 2026	M	69	Head	EUS-FNB	*KRAS A164V*, *BRAF G469V*	RT	NED

Abbreviations: FU: follow-up, F: female, NA: not available, RT: radiotherapy, DOD: dead of disease, M: male, NED: no evidence of disease, FNA: fine-needle aspiration, CT: chemotherapy, EUS: endoscopic ultrasound, IHC: immunohistochemistry, AWD: alive with disease, FNB: fine-needle biopsy.

## Data Availability

All data generated or analyzed during this study are included in this published article.

## Supplementary Materials

The following supporting information can be downloaded at https://www.mdpi.com/article/10.3390/diagnostics16172680/s1, Table S1: List of antibodies used in the present series.

## Abbreviations

The following abbreviations are used in this manuscript:

SPB	Solitary plasmacytoma of the bone
EMP	Extramedullary plasmacytoma
EUS	Endoscopic ultrasound
FNB	Fine-needle biopsy
TA	Tissue acquisition
NGS	Next-generation sequencing
FDG-PET	Fluorodeoxyglucose positron emission tomography
FNA	Fine-needle aspiration
EMD	Extraosseous extramedullary disease
EMZL	Extranodal marginal zone lymphoma
LPL	Lymphoplasmacytic lymphoma
