# Supplementary material for: Solitary Pancreatic Extramedullary Plasmacytoma Diagnosed Through Endoscopic Ultrasound-Guided Fine-Needle Biopsy—The Role of Endoscopic Diagnosis and Multidisciplinary Approach: A Case Report and Literature Review
Source: Diagnostics (Basel). 2026 Aug 22;16(17):2680. doi: 10.3390/diagnostics16172680 (PMC13565103; doi:10.3390/diagnostics16172680)
Supplement: Supplementary file 1 [file diagnostics-16-02680-s001.zip › diagnostics-4470425-supplementary.pdf]

Table S1. List of antibodies used in the present series.

Antibody	Clone	Provider
CD138	B-A38	Cell Marque
MUM1	EP190	Roche
Kappa	Polyclonal	Roche
Lambda	Polyclonal	Roche
Pan-cytokeratin	AE1/AE3/PCK26	Roche
Cytokeratin 7	SP52	Roche
CD45	2B11	Cell Marque
S-100	4C4.9	Roche
SOX10	SP267	Roche
Synaptophysin	SP11	Roche
Chromogranin-A	LK2H10	Roche
CD68	KP1	Santa Cruz
CD34	QBEnd/10	Roche
Desmin	DE-R-11	Roche
HMB45	HMB45	Roche
CD20	L26	Roche
Cyclin D1	SP4-R	Roche
CD56	MRQ-42	Roche
Ki67	30-9	Ventana
